# Combined use of low T3 syndrome and NT-proBNP as predictors for death in patients with acute decompensated heart failure

**DOI:** 10.1186/s12902-021-00801-x

**Published:** 2021-07-02

**Authors:** Xinke Zhao, Rongcheng Zhang, Hugang Jiang, Kai Liu, Chengxu Ma, Ming Bai, Tao An, Younan Yao, Xinqiang Wang, Ming Wang, Yingdong Li, Yuhui Zhang, Jian Zhang

**Affiliations:** 1 Heart Failure Center, Fuwai Hospital, National Center for Cardiovascular Diseases, Chinese Academy of Medical Sciences & Peking Union Medical College, 167 Beilishi Road, 100037 Beijing, China; 2 Department of Cardiology, Affiliated Hospital of Gansu University of Chinese Medicine, 732 Jiyuguanxi Road, 730000 Lanzhou, China; 3 Department of Cardiology, The First Hospital of Lanzhou University, 730000 Lanzhou, China

**Keywords:** Acute decompensated heart failure, Low T3 syndrome, NT-proBNP, Mortality

## Abstract

**Background:**

In patients with established HF, low triiodothyronine syndrome (LT3S) is commonly present, and N-terminal pro-B-type natriuretic peptide (NT-proBNP) is a useful marker for predicting death. This study was aimed to evaluate the prognostic value of LT3S in combination with NT-proBNP for risk of death in patients with heart failure (HF).

**Methods:**

A total of 594 euthyroid patients hospitalized with acute decompensated HF were enrolled by design. Of these patients, 27 patients died during hospitalization and 100 deaths were identified in patients discharged alive during one year follow-up. Patients were divided into 2 groups on the base of the reference ranges of free T3 (FT3) levels: LT3S group (FT3 < 2.3pg/mL, n = 168) and non-LT3S group (FT3 ≥ 2.3pg/mL, *n* = 426).

**Results:**

In multivariable Cox regression, LT3S was significantly associated with 1 year all-cause mortality (adjusted hazard ratio, 1.85; 95 % confidence interval [CI], 1.21 to 2.82; *P* = 0.005), but not significant for in-hospital mortality (adjusted hazard ratio, 1.58; 95 % CI, 1.58 to 2.82; *P* = 0.290) after adjustment for clinical variables and NT-proBNP.

Addition of LT3S and NT-proBNP to the prediction model with clinical variables significantly improved the C statistic for predicting 1 year all-cause mortality.

**Conclusions:**

In patients with acute decompensated HF, the combination of LT3S and NT-proBNP improved prediction for 1 year all-cause mortality beyond established risk factors, but was not strong enough for in-hospital mortality.

**Supplementary Information:**

The online version contains supplementary material available at 10.1186/s12902-021-00801-x.

## Background

Patients with heart failure (HF), especially those with advanced disease status, are more likely to experience neurohormonal derangements which could reflect the pathophysiological process and predict the deterioration of HF [1]. Thyroid hormone metabolic abnormality is one of the important phenotype of hormonal disorders in patients with HF [2]. Low T3 syndrome (LT3S) that refers to reduced triiodothyronine (T3) with normal levels of thyroid stimulating hormone (TSH) and tetraiodothyronine (T4) is commonly present in patients with established HF, and accounts for about half the number of euthyroid patients with acute HF [3–6]. The low level of free T3 (FT3) in LT3S has been documented to be an independent risk predictors in patients with HF [2]. Given well-establishment of N-terminal pro-B-type natriuretic peptide (NT-proBNP) in patients with HF, it remains unclear how to use of LT3S in combination with NT-proBNP for risk prediction in patients with acute decompensated HF. Furthermore, data on the evaluation of prognostic value of LT3S in HF patients in short term are lacking. Therefore, the purpose of this study was to investigate the prognostic value of LT3S by the use of FT3, and in combination with NT-proBNP as predictors for in-hospital mortality and 1-year all-cause mortality in euthyroid patients hospitalized with acute decompensated HF.

## Methods

### Study population and design

From December 2017 to January 2019, we prospectively performed an observational study for patients hospitalized with acute decompensated HF in two hospitals (Affiliated Hospital of Gansu University of Chinese Medicine and the First Hospital of Lanzhou University) from Gansu province, China. Patients who were aged 18 years or older, gave written informed consent, and had thyroid function and NT-proBNP available from routine laboratory measurements were consecutively included. Acute decompensated HF was diagnosed by at least two cardiologists according to guideline [7] and defined as worsening of signs and symptoms of preexisting HF resulting in unplanned hospitalization. Patients with a diagnosis of acute coronary syndrome, cancer, autoimmune disease, and previous thyroid disease (history of thyroid dysfunction, treatment with thyroid hormones or anti-thyroid drugs, and previous thyroid operation or radiation therapy) were excluded. Patients were also excluded if they were prescribed amiodarone before admission. All patients were administered intravenous loop diuretics during the first 24 h of admission. Information on the study population including demographic characteristics, comorbidities, vital signs, physical examination, New York Heart Association (NYHA) functional class and medication history are recorded by trained clinicians at admission. Laboratory data of patients were collected within 24 h of admission. Echocardiography was blindly performed on all patients by specialists trained in cardiac ultrasonography during hospitalization. All clinical data were entered into a predefined case report form by an abstractor with checking by another. According to the results of measurement of thyroid function, patients with euthyroidism (TSH and free T4 in the normal reference ranges) were selected for analysis and divided into 2 groups on the base of the reference ranges of FT3: LT3S group and non-LT3S group. In-hospital death and 1-year all-cause death were obtained from patients’ medical records or by contact with patients or patients’ families. The study protocol was in compliance with the Declaration of Helsinki, and the ethics committee of Affiliated Hospital of Gansu University of Chinese Medicine and the First Hospital of Lanzhou University approved this study (approve number: 2017-04).

### Measurement of thyroid function and NT-proBNP

Thyroid function including TSH, free T4, total T4, FT3, and total T3 was measured by ADVIA Centaur XP Chemiluminescent Immunoassay System (Siemens AG, Munich, Germany) in clinical laboratory. The reference ranges for FT3 were 2.3 to 4.2 pg/mL.

NT-proBNP was measured by the electrochemiluminescence immunoassay using the Elecsys 2010 analyser (Roche Diagnostics, Mannheim, Germany). Laboratory technicians who performed biomarkers measurement at Affiliated Hospital of Gansu University of Chinese Medicine and the First Hospital of Lanzhou University were blinded to this study.

### Statistical Analyses

We tested the normality of continuous variables by using Kolmogorov-Smironov test. Continuous variables are presented as means ± standard deviation (SD) or medians and interquartile range (IQR). Categorical variables are described as numbers and percentages. Comparisons between two groups were performed by Student t-test for symmetrical continuous, Mann-Whitney U test for nonsymmetric continuous, and χ2 tests for categorical variables. Logarithmic transformation was performed to normalize the distribution of NT-proBNP. Candidate variables that were associated with LT3S in univariate logistic regression (*P* < 0.05) were included in multiple logistic regression for explaining the variability of LT3S. One-year mortality was assessed in patients discharged alive. Cox regression was performed to evaluate the associations of LT3S and NT-proBNP with risk of death. Candidate variables (without NT-proBNP) that was associated with death on univariable Cox regression analysis (*P* < 0.05) were retained in the multiple Cox regression analysis. Variables with significant *P* values (*P* < 0.05) were retained in the final multivariable model. Hosmer-Lemeshow statistic was used to evaluate model calibration. The additional values of LT3S and NT-proBNP to reference model for detection of death were evaluated using Harrell’s C-statistic. Log-rank tests for Kaplan-Meier cumulative hazard curves were used to describe the ability of LT3S to predict death. *P* value of less than 0.05 from two-sided tests was considered statistically significant. All analyses were conducted using SPSS version 22.0 (SPSS Inc., Chicago, Illinois) and Stata version 15.1 (StataCorp LP, College Station, TX, USA).

## Results

**Fig. 1:**
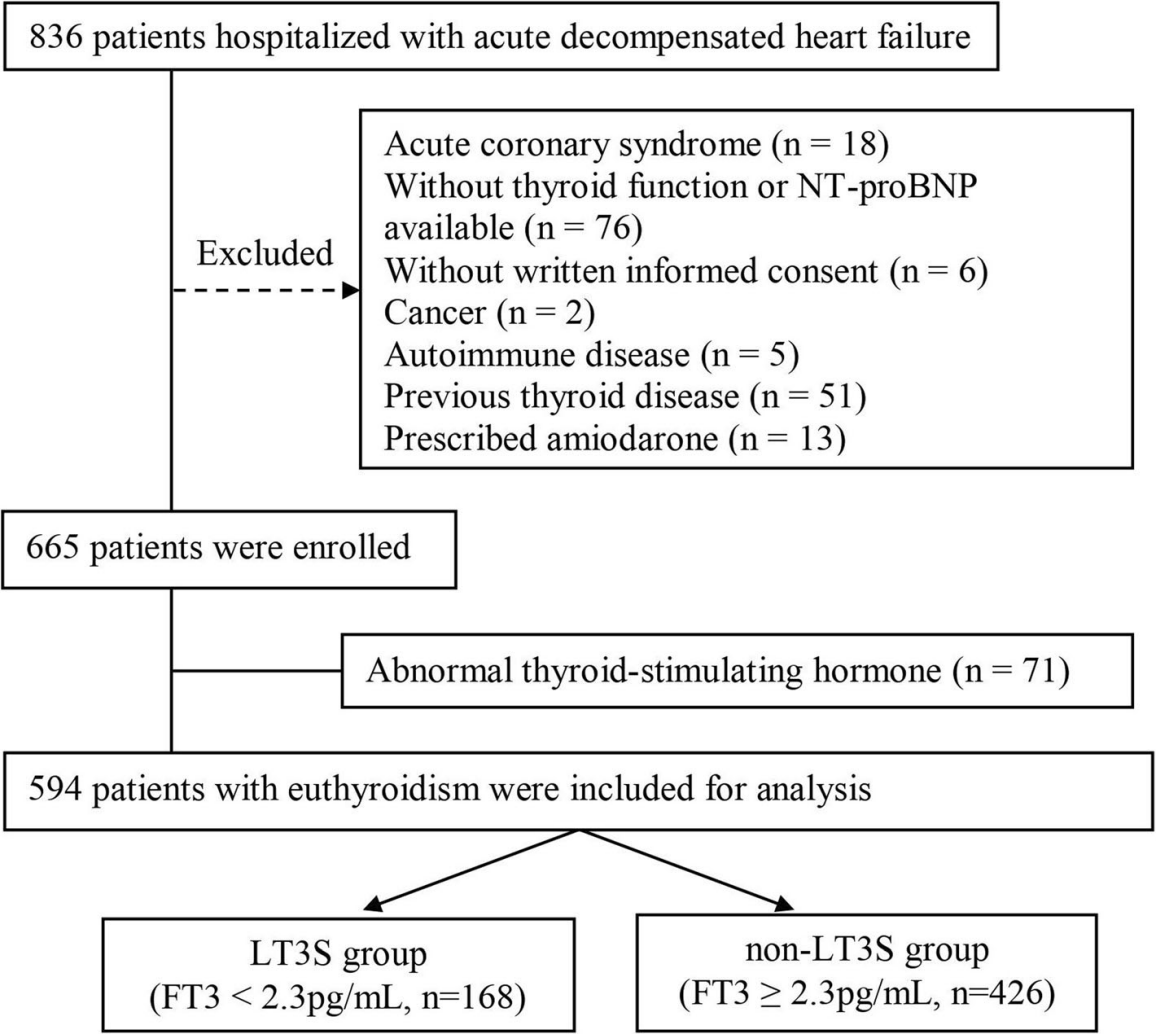
Flow chart for patient selection. LT3S: low T3 syndrome

Of the 665 patients included from December 2017 to January 2019, 594 patients with euthyroidism were included for analysis (Fig. 1). The mean age was 57 years, 414 (69.7 %) patients were male and 317 (53.4 %) patients had LVEF < 40 %.

In this study, one hundred and sixty eight (28.3 %) patients had LT3S (FT3 < 2.3 pg/mL). Comparisons of baseline characteristics according to patients with LT3S and non-LT3S (FT3 ≥ 2.3pg/mL) are presented in Table 1. Patients with LT3S were more likely to be older, with higher atrial fibrillation proportion, more advanced NYHA class, and lower systolic blood pressure and body mass index; in addition, intravenous infusion of dopamine was used more frequently in patients with LT3S. In multivariable logistic regression analysis, independent determinants of LT3S were atrial fibrillation, systolic blood pressure, NYHA functional class, hemoglobin, albumin, and blood urea nitrogen (Supplemental Table 1).

**Table 1:** Baseline characteristics of patients according to FT3 calssification

Variable	LT3S group (*n* = 168)	non-LT3S group (*n* = 426)	*P* value
Age, year	61 ± 16	57 ± 15	0.009
Male, n (%)	114 (67.9)	300 (70.4)	0.540
History, n (%)			
Hypertension	80(47.6)	189(44.4)	0.473
Diabetes mellitus	44(26.2)	88(20.7)	0.144
Ischemic heart disease	58(34.5)	142(33.3)	0.782
Dilated cardiomyopathy	38(22.6)	118(27.7)	0.205
Valvular heart disease	47(28.0)	98(23.0)	0.204
Atrial fibrillation	82(49.1)	134(32.2)	< 0.001
Physical examination			
Heart rate, beats/min	79 ± 19	79 ± 16	0.999
Systolic blood pressure, mmHg	112 ± 19	117 ± 19	0.003
Body mass index, kg/m^2^	23.0 ± 4.3	24.0 ± 4.7	0.016
NYHA functional class, n (%)			< 0.001
II	11(6.5)	86(20.2)	
III	68(40.5)	204(47.9)	
IV	89(53.0)	136(31.9)	
LVEF (%)	41.6 ± 14.1	39.6 ± 14.5	0.128
LVEF < 40 %, n (%)	82(48.8)	235(55.2)	0.162
LVEDD, mm	60.0 ± 13.2	62.5 ± 12.8	0.031
Laboratory results			
Hemoglobin, g/dL	126.1 ± 26.4	138.5 ± 20.8	< 0.001
Sodium, mmol/L	138.2 ± 4.2	139.6 ± 3.3	< 0.001
Albumin, g/dL	37.4 ± 4.8	40.5 ± 4.4	< 0.001
Blood urea nitrogen, mmol/L	11.5 ± 6.5	8.4 ± 3.9	< 0.001
Creatinine, umol/L	117.1 ± 57.7	96.8 ± 36.3	< 0.001
NT-proBNP, pg/mL	3165(1441–5525)	1756(998–3263)	< 0.001
Medication on presentation, n (%)			
Diuretics	134(79.8)	330(77.5)	0.542
ACEI/ARB/ARNI	99(58.9)	236(55.4)	0.435
β-blockers	132(78.6)	328(77.0)	0.679
Spironolactone	106(63.1)	262(61.5)	0.719
Intravenous cardiotonic therapy			
Dopamine	121(72.0)	191(44.8)	< 0.001
Dobutamine	5(3.0)	13(3.1)	0.961
Norepinephrine	7(4.2)	16(3.8)	0.815
In-hospital death, n (%)	15(8.9)	12(2.8)	0.001
One-year all cause death, n (%)^a^	53(34.6)	47(11.3)	< 0.001

^a^For patients discharged alive. *ACEI* angiotension-converting enzyme inhibitor; *ARB* angiotensin receptor blocker; *eGFR* estimated glomerular filtration rate; *FT3* free triiodothyronine; *LVDD* left ventricular diastolic diameter; *LVEF* left ventricular ejection fraction; *NT-proBNP* N-terminal pro-B-type natriuretic peptide; *NYHA* New York Heart Association

In all, 27 patients (4.5 %) died during hospitalization. Compared with patient with non-LT3S (2.8 %), patients with LT3S had a higher proportion of in-hospital mortality (8.9 %) (Table 1). Results of univariable analysis for all potential risk predictors and multivariable models for in-hospital mortality are shown in supplemental Tables 2 and supplemental Table 4. NYHA functional class and blood urea nitrogen were included as model for in-hospital mortality. In univariable Cox regression analysis, patients with LT3S were significantly associated with in-hospital mortality compared with patient with non-LT3S, but the association was not significant in multivariable analysis (Table 2). Incorporation of both LT3S and NT-proBNP to reference model could not improve C statistic for in-hospital mortality (Table 3).

**Table 2:** Association of LT3S and NT-proBNP with risk of death

	In-hospital death		One year all-cause death	
	HR(95 % CI)	*P* value	HR(95 % CI)	*P* value
**LT3S**				
Unadjusted	3.382(1.548–7.389)	0.002	3.554(2.399–5.265)	< 0.001
Model^a^	1.872(0.813–4.309)	0.140	1.928(1.264–2.941)	0.002
Model^a^ + Log (NT-proBNP)	1.583(0.676–3.708)	0.290	1.845 (1.206–2.823)	0.005
**Log (NT-proBNP)**				
Unadjusted	2.861(1.744–4.692)	< 0.001	2.572(2.017–3.280)	< 0.001
Model^a^	2.071(1.209–3.546)	0.008	1.919(1.456–2.529)	< 0.001
Model^a^+ LT3S	1.983(1.151–3.416)	0.014	1.891(1.430–2.500)	< 0.001

^a^Model for in-hospital death: blood urea nitrogen and New York Heart Association functional class

Model for one-year all-cause death: blood urea nitrogen, systolic blood pressure, body mass index, New York Heart Association functional class, sodium, and albumin. *LT3S* low T3 syndrome; *NT-proBNP* N-terminal pro-B-type natriuretic peptide

**Table 3:** C statistic for models predicting in-hospital mortality and 1-year all-cause mortality in HF patients with euthyroidism

	In-hospital death			1-year all-cause death		
	**C statistic** **(95 % CI)**	***P*value**	**H-L** ***P*value**	**C statistic** **(95 % CI)**	***P*value**	**H-L** ***P*value**
Model*	0.789 (0.717–0.862)	Reference	0.765	0.783 (0.737–0.830)	Reference	
Model* + Log (NT-proBNP)	0.830 (0.752–0.905)	0.092	0.883	0.805 (0.758–0.852)	0.126	0.507
Model* + LT3S	0.800 (0.728–0.872)	0.489†	0.954	0.793 (0.746–0.840)	0.297†	0.697
Model* + two variables	0.833(0.753–0.908)	0.088†	0.382	0.813 (0.768–0.859)	0.047†	0.996

*Model for in-hospital mortality: blood urea nitrogen and New York Heart Association functional class. Model for 1 year all-cause mortality: blood urea nitrogen, systolic blood pressure, body mass index, New York Heart Association functional class, sodium, and albumin. †*P* > 0.05 when compared with Model + NT-proBNP. *H-L* Hosmer-Lemeshow statistic; *LT3S* low T3 syndrome; *NT-proBNP* N-terminal pro-B-type natriuretic peptide

In 567 patients discharged alive, 100 deaths (17.6 %) were identified during one year follow-up. Patients with LT3S had a higher proportion of 1 year all-cause mortality (34.6 %) than that in patient with non-LT3S (11.3 %) (Table 1). Results of univariable analysis for all potential risk predictors and multivariable models for 1 year all-cause mortality are shown in supplemental Tables 3 and supplemental Table 4. Model for 1-year all-cause mortality included systolic blood pressure, body mass index, NYHA functional class, sodium, albumin, and blood urea nitrogen. In univariable Cox regression analysis, patients with LT3S were significantly associated with 1-year all-cause mortality compared with patient with non-LT3S, this association remained significant when adjusted for reference model with or without NT-proBNP included (Table 2). Incorporation of both LT3S and NT-proBNP to reference model could improve C statistic for 1-year all-cause mortality (Table 3).

**Fig. 2:**
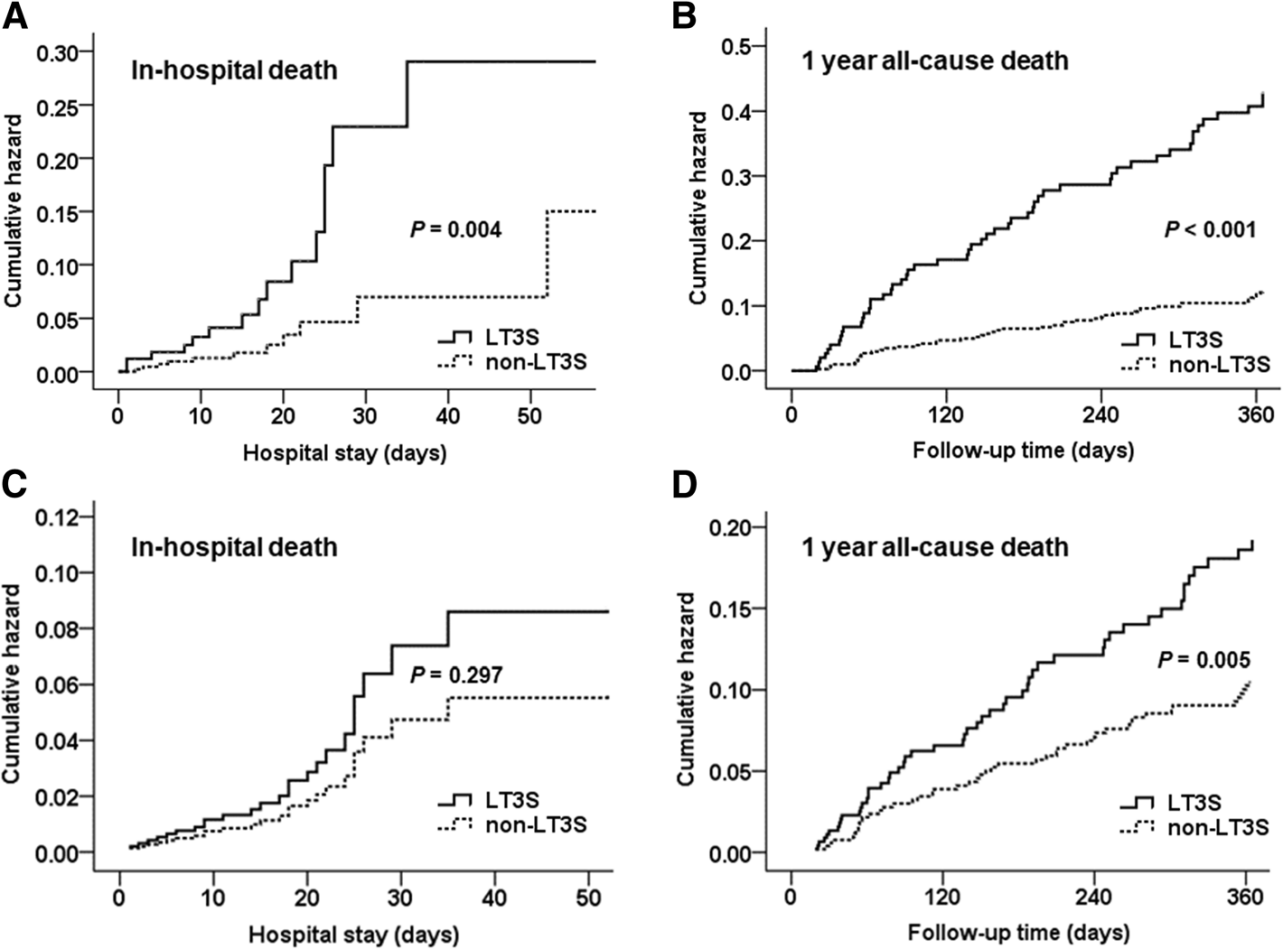
Kaplan–Meier curves for death according to patients with low T3 syndrome (LT3S) and non- LT3S. Kaplan–Meier curves for (**A**) in-hospital mortality and (**B**) 1 year all-cause mortality. Adjusted Kaplan-Meier curves by New York Heart Association (NYHA) functional class, blood urea nitrogen and NT-proBNP for (**C**) in-hospital mortality, and by systolic blood pressure, body mass index, NYHA functional class, sodium, albumin, blood urea nitrogen, and NT-proBNP for (**D**) 1 year all-cause mortality

Kaplan-Meier cumulative hazard curves showed the association of LT3S with risk of death compared with patients with non-LT3S (Fig. 2).

## Discussion

This study showed 28.3 % of euthyroid patients with acute decompensated HF had LT3S. In multiple Cox regression, LT3 syndrome did not remain significant adjusted hazard ratio for in hospital mortality but did remain in the model next to NTproBNP for 1-year all-cause mortality. As for in-hospital mortality prediction, LT3S only had a significant unadjusted hazard ratio, and the combination of LT3S and NT-proBNP for risk prediction was not strong enough.

LT3S is an important phenotype among HF patients with thyroid disorders. In our study, 28.3 % of euthyroid patients hospitalized with acute decompensated HF presented with LT3S, which was similar to the report of 15.3–53.5 % by previous studies [3–6, 8]. The mechanisms underlying this disorder may be attributable to multiple factors including decreased T4 transportation into tissues [9], impaired T4 to T3 conversion result from diminished activity of phenolic ring deiodinase (type I and type II deiodinase) [10–12], increased inactivation of T4 and T3 associated with increasing activity of tyrosil ring deiodinase (type III deiodinase) [10, 13] and impairment of TRH metabolism [13, 14]. In multivariable logistic regression analysis, we found that variables, which partially reflect the status of nutrition (hemoglobin, albumin), liver (albumin) and kidney function (blood urea nitrogen), and volume overload (NYHA functional class, systolic blood pressure), have a significant effect on LT3S. Malnutrition and impairment of liver and kidney has been documented to be associated with reduced T3 through decreasing type I deiodinase activity [5], which predominantly expressed in the liver and kidney and is responsible for most of circulating T3 levels [15, 16]. In addition, volume overload could lead to circulation disorder and tissue hypoxia and result in inactivation of T3 through induction of type III deiodinase activity [2, 13]. Notably, age was not independently associated with LT3S in our results. In contrast, a recent report in 956 euthyroid patients with acute HF suggested an independent association between LT3S and aging [6]. These difference are probably explained by the lower mean age of patients in our study (57 years old vs. 70 years old). The different proportion of comorbid conditions such as ischemic heart disease and hypertension might possible cause such regional characteristics [17]. Consistent with previous reports [6], we also found that patients with LT3S are more likely to receive intravenous infusion of dopamine. The use of inotropics might have the potential to increase FT3 values and is associated with short-term hemodynamic and neurohormonal improvement [18]. Further studies are needed to validate the implication of inotropics treatment in patients with LT3S.

Generally, LT3S was considered an adaptive mechanism to reduce metabolic demand in early phase of HF, and a maladaptive mechanism to be associated with comorbidities when decompensated HF occurs and progresses [19]. Several studies have reported a weak association between T3 and LVEF in HF patient with or without severe symptom [5, 6, 20]. The involvement of T3 in comorbidities of patients with decompensated HF might represent a potential advantage for prognostic value over established biomarkers such as natriuretic peptides, which have been validated to be important markers for left ventricular dysfunction. Although previous published reports have demonstrating that low T3 was associated with worse outcomes in patients with acute HF during hospitalization [4] and long term follow-up [4–6, 8, 21], data about the combination of LT3S and NT-proBNP as risk predictors was limited. A study by Chuang et al. showed that total T3 remained as predictor of prognosis for mortality beyond clinical risk factor and NT-proBNP, while free T3 was not predictive in univariate analysis in 106 critically ill patients with acute HF [21]. The weak result for free T3 are probably due to the lower FT3 levels in patients with severe condition and older age (mean age was 71 years), smaller sample size, and a broader spectrum of follow-up time (including short and longer term follow-up).

With respect to prognosis during hospitalization, Rothberger et al. have reported that FT3 was significantly associated with length of stay in adjusted model, but failed to find association between FT3 and hospital death because only 3 in-hospital deaths were identified [4]. In the present study, we found the combination of LT3S and NT-proBNP in predicting in-hospital mortality was not strong enough. This could be explained that LT3S was probably reversed by inotropic stimulation, the number of events of in hospital mortality was small, and the presence of LT3S might be a chronic pathophysiologic process in the progression of HF. Furthermore, FT3, as a marker of multi-system impairment, could not directly reflect left ventricular dysfunction and temporal hemodynamic parameters [5]. Further studies with serial measurement of FT3 in euthyroid patients with decompensated HF will be helpful to validate this statement and to identify the association between FT3 changes and HF progression.

Our study has several limitations. First, in this observational study, the potential efficacy of T3 replacement in patients with LT3S was not evaluated. Second, although logistic regression analysis had detect the variables affecting LT3S in this study, we did not deeply understand the process of LT3S during therapy and follow-up by monitoring the change of FT3, especially for those without LT3S at admission. Third, clinical data at baseline was used to identify risk factors for patients discharged alive. These factors might not be able to better reflect the status of patients at discharge. Other factors such as invasive therapies, discharge instruction and post-discharge management might affect the results.

## Conclusions

This study firstly investigated the prognostic value of LT3S and NT-proBNP as predictors for mortality in patients hospitalized with acute decompensated HF in short and longer term. The combination of LT3S and NT-proBNP was effective for improving risk stratification and discrimination for 1 year all-cause mortality, but was weak to provide additional prognostic value for in-hospital mortality. The results of this study will help clinicians more accurately assess the risk of patients with decompensated HF and tailor their therapies.

## Supplementary Information

**Additional file 1.** 

**Additional file 2.**

**Additional file 3.**

**Additional file 4.**

## Data Availability

The datasets used and analysed during the current study can be obtained from the corresponding author on reasonable request.

## Abbreviations

ACEI: Angiotension-converting enzyme inhibitor
ARB: Angiotensin receptor blocker
eGFR: Estimated glomerular filtration rate
FT3: Free triiodothyronine
HF: :Heart failure
H-L: Hosmer-Lemeshow statistic
LT3S: Low T3 syndrome
LVDD: Left ventricular diastolic diameter
LVEF: Left ventricular ejection fraction
NT-proBNP: N-terminal pro-B-type natriuretic peptide
NYHA: New York Heart Association
ROC: Receiver operating characteristic
T4: Tetraiodothyronine
TSH: Thyroid stimulating hormone
