# Supplementary material for: Combined use of low T3 syndrome and NT-proBNP as predictors for death in patients with acute decompensated heart failure
Source: BMC Endocr Disord. 2021 Jul 2;21:140. doi: 10.1186/s12902-021-00801-x (PMC8252209; doi:10.1186/s12902-021-00801-x)
Supplement: Supplementary file 1 — Additional file 1. [file 12902_2021_801_MOESM1_ESM.docx]

| **Supplemental Table 1.** Univariable and multivariable Logistic regression analysis for predicting low T3 syndrome | | | | | |
| --- | --- | --- | --- | --- | --- |
| Variable | Univariable | |  | Multivariable | |
|  | HR (95% CI) | *P* value |  | HR (95% CI) | *P* value |
| Sex, male | 0.887(0.603-1.303) | 0.540 |  | - | - |
| Age, year | 1.017(1.004-1.029) | 0.010 |  | - | - |
| Diabetes mellitus | 1.363(0.899-2.067) | 0.145 |  | - | - |
| Atrial fibrillation | 0.493(0.341-0.711) | <0.001 |  | 1.673(1.093-2.561) | 0.018 |
| Body mass index, kg/m^2^ | 0.945(0.903-0.989) | 0.015 |  | - | - |
| Heart rate, beats/min | 1.000(0.989-1.011) | 0.999 |  | - | - |
| Systolic blood pressure, mmHg | 0.985(0.975-0.995) | 0.003 |  | 0.983(0.971-0.995) | 0.007 |
| NYHA functional class | 2.137(1.613-2.830) | <0.001 |  | 1.642(1.183-2.281) | 0.003 |
| Left ventricular ejection fraction (%) | 1.010(0.997-1.022) | 0.128 |  | - | - |
| Sodium, mmol/L | 0.903(0.858-0.950) | <0.001 |  | - | - |
| Hemoglobin, g/dL | 0.976(0.968-0.984） | <0.001 |  | 0.983(0.974-0.993) | 0.001 |
| Albumin, g/dL | 0.860(0.823-0.898) | <0.001 |  | 0.908(0.863-0.955) | <0.001 |
| Blood urea nitrogen, mmol/L | 1.141(1.094-1.190) | <0.001 |  | 1.078(1.017-1.143) | 0.012 |
| Creatinine, umol/L | 1.010(1.006-1.014) | <0.001 |  | - | - |
| Log (NT-proBNP) | 1.904(1.520-2.385) | <0.001 |  | - | - |

NT-proBNP = N-terminal pro-B-type natriuretic peptide; NYHA = New York Heart Association
