# Supplementary material for: Combined use of low T3 syndrome and NT-proBNP as predictors for death in patients with acute decompensated heart failure
Source: BMC Endocr Disord. 2021 Jul 2;21:140. doi: 10.1186/s12902-021-00801-x (PMC8252209; doi:10.1186/s12902-021-00801-x)
Supplement: Supplementary file 2 — Additional file 2. [file 12902_2021_801_MOESM2_ESM.docx]

| **Supplemental Table 2.** Univariable and multivariable Cox regression analysis for predicting in-hospital mortality | | | | | |
| --- | --- | --- | --- | --- | --- |
| Variable | Univariable | |  | Multivariable | |
|  | HR (95% CI) | *P* value |  | HR (95% CI) | *P* value |
| Age, year | 0.998(0.973-1.024) | 0.897 |  | - | - |
| Male | 1.061(0.461-2.443) | 0.889 |  | - | - |
| Hypertension | 0.823(0.375-1.806) | 0.628 |  |  |  |
| Diabetes mellitus | 0.741(0.255-2.154) | 0.582 |  | - | - |
| Ischemic heart disease | 0.339(0.117-0.985) | 0.047 |  | - | - |
| Atrial fibrillation | 1.722(0.797-3.720) | 0.167 |  | - | - |
| Heart rate, beats/min | 1.023(1.002-1.045) | 0.028 |  | - | - |
| Systolic blood pressure, mmHg | 0.970(0.948-0.993) | 0.011 |  | - | - |
| Body mass index, kg/m^2^ | 0.950(0.860-1.049) | 0.312 |  | - | - |
| NYHA functional class | 6.522(2.573-16.531) | <0.001 |  | 4.544(1.710-12.079) | 0.002 |
| Left ventricular ejection fraction (%) | 1.011(0.984-1.038) | 0.431 |  | - | - |
| Hemoglobin, g/dL | 0.989(0.973-1.005) | 0.165 |  | - | - |
| Sodium, mmol/L | 0.859(0.779-0.947) | 0.002 |  | - | - |
| Albumin, g/dL | 0.879(0.818-0.945) | <0.001 |  | - | - |
| Blood urea nitrogen, mmol/L | 1.096(1.044-1.152) | <0.001 |  | 1.074(1.017-1.134) | 0.010 |
| Creatinine, umol/L | 1.004(0.997-1.011) | 0.231 |  | - | - |

NYHA = New York Heart Association
