# Supplementary material for: Combined use of low T3 syndrome and NT-proBNP as predictors for death in patients with acute decompensated heart failure
Source: BMC Endocr Disord. 2021 Jul 2;21:140. doi: 10.1186/s12902-021-00801-x (PMC8252209; doi:10.1186/s12902-021-00801-x)
Supplement: Supplementary file 3 — Additional file 3. [file 12902_2021_801_MOESM3_ESM.docx]

| **Supplemental Table 3.** Univariable and multivariable Cox regression analysis for predicting 1 year all-cause mortality in patients discharged alive | | | | | |
| --- | --- | --- | --- | --- | --- |
| Variable | Univariable | |  | Multivariable | |
|  | HR (95% CI) | *P* value |  | HR (95% CI) | *P* value |
| Age, year | 1.019(1.004-1.034) | 0.011 |  | - | - |
| Male | 0.787(0.503-1.230) | 0.293 |  | - | - |
| Hypertension | 0.791(0.530-1.181) | 0.252 |  |  |  |
| Diabetes mellitus | 1.027(0.645-1.636) | 0.911 |  | - | - |
| Ischemic heart disease | 1.140(0.760-1.711) | 0.527 |  | - | - |
| Atrial fibrillation | 1.765(1.193-2.613) | 0.004 |  | - | - |
| Heart rate, beats/min | 0.999(0.986-1.011) | 0.815 |  | - | - |
| Systolic blood pressure, mmHg | 0.980(0.969-0.991) | <0.001 |  | 0.987(0.977-0.998) | 0.018 |
| Body mass index, kg/m^2^ | 0.885(0.839-0.934) | <0.001 |  | 0.936(0.891-0.984) | 0.010 |
| NYHA functional class | 2.051(1.499-2.808) | <0.001 |  | 1.979(1.452-2.696) | <0.001 |
| Left ventricular ejection fraction (%) | 0.998(0.984-1.012) | 0.769 |  | - | - |
| Hemoglobin, g/dL | 0.990(0.981-0.998) | 0.018 |  | - | - |
| Sodium, mmol/L | 0.901(0.857-0.947) | <0.001 |  | 0.938(0.893-0.987) | 0.013 |
| Albumin, g/dL | 0.921(0.887-0.955) | <0.001 |  | 0.951(0.919-0.984) | 0.004 |
| Blood urea nitrogen, mmol/L | 1.111(1.082-1.141) | <0.001 |  | 1.073(1.027-1.122) | 0.002 |
| Creatinine, umol/L | 1.009(1.006-1.013) | <0.001 |  | - | - |

NYHA = New York Heart Association
