# Supplementary material for: Combined use of low T3 syndrome and NT-proBNP as predictors for death in patients with acute decompensated heart failure
Source: BMC Endocr Disord. 2021 Jul 2;21:140. doi: 10.1186/s12902-021-00801-x (PMC8252209; doi:10.1186/s12902-021-00801-x)
Supplement: Supplementary file 4 — Additional file 4. [file 12902_2021_801_MOESM4_ESM.docx]

| **Supplemental Table 4.** Multivariable Cox regression analysis for predicting death in the final model | | | | | |
| --- | --- | --- | --- | --- | --- |
| Variable | In–hospital death | |  | One year all-cause death | |
|  | HR (95% CI) | *P* value |  | HR (95% CI) | *P* value |
| Systolic blood pressure, mmHg | - | - |  | 0.990(0.979-1.002) | 0.093 |
| Body mass index, kg/m^2^ | - | - |  | 0.941(0.889-0.997) | 0.038 |
| NYHA functional class | 5.163(1.967-13.549) | 0.001 |  | 1.446(1.021-2.047) | 0.038 |
| Sodium, mmol/L | - | - |  | 0.937(0.884-0.993) | 0.029 |
| Albumin, g/dL | - | - |  | 0.980(0.939-1.022) | 0.335 |
| Blood urea nitrogen, mmol/L | 1.046(0.988-1.108) | 0.122 |  | 1.060(1.027-1.094) | <0.001 |
| Log (NT-proBNP) | 1.983(1.151-3.416) | 0.014 |  | 1.891(1.430-2.500) | <0.001 |
| Low T3 syndrome | 1.583(0.676-3.708) | 0.290 |  | 1.845 (1.206-2.823) | 0.005 |

HR=Hazard ratios; NT-proBNP=N-terminal pro-B-type natriuretic peptide; NYHA = New York Heart

Association
